# Supplementary material for: Observation of interface piezoelectricity in superconducting devices on silicon
Source: Nat Commun. 2025 Dec 5;17:377. doi: 10.1038/s41467-025-67066-z (PMC12796224; doi:10.1038/s41467-025-67066-z)
Supplement: Supplementary file 1 — Supplementary Information [file 41467_2025_67066_MOESM1_ESM.pdf]

Supplementary Information for "Observation of interface piezoelectricity in superconducting devices on silicon"

Haoxin Zhou,[1,2,3] Eric Li,[1,5,*] Kadircan Godeneli,[1,2] Zi-Huai Zhang,[1,2,3] Shahin Jahanbani,[3,2] Kangdi Yu,[1,2] Mutasem Odeh,[1,2] Shaul Aloni,[4] Sinéad Griffin,[2,4] and Alp Sipahigil[1,2,3,†]

[1] *Department of Electrical Engineering and Computer Sciences, University of California, Berkeley, Berkeley, California 94720, USA*
[2] *Materials Sciences Division, Lawrence Berkeley National Laboratory, Berkeley, California 94720, USA*
[3] *Department of Physics, University of California, Berkeley, Berkeley, California 94720, USA*
[4] *Molecular Foundry, Lawrence Berkeley National Laboratory, Berkeley, California 94720, USA*
[5] *Department of Electrical Engineering and Computer Science, Massachusetts Institute of Technology, Cambridge, MA 02139*

This PDF file includes

- Interference between crosstalk and piezoelectricity-induced microwave transmission
- Extracting K^2 from microwave transmission coefficient
- Error analysis of K^2_{eff}
- Qubit loss rate induced by the interface piezoelectricity
- Supplementary table and figures

Interference between crosstalk and piezoelectricity-induced microwave transmission

The transmission coefficient S_{21} of the IDTs is defined to be

$$S_{21}(\omega) = \frac{V_2^-}{V_1^+}, \tag{S1}$$

where V_1^+ is the voltage wave propagating into the launching IDT, V_2^- is the voltage wave propagating out of the detecting IDT, and $\omega = 2\pi f$ is the angular frequency.

When a steady drive $V_1 = V_1^+ + V_1^- = |V_1|e^{i(\omega t+\phi_1)}$ is applied to the launching IDT, the forward-propagating voltage of the detecting IDT will have the form

$$V_2^- = \left(V_e^+(\omega, V_1)e^{i\omega d/v_e} + V_a^+(\omega, V_1)e^{i\omega d/v_a}\right)e^{i\omega t}, \tag{S2}$$

where $V_e^+(V_1,\omega)$ and $V_a^+(V_1,\omega)$ are the amplitudes of the forward-propagating voltage waves induced by electromagnetic crosstalk and interface piezoelectricity, respectively. v_e and v_a are the speeds of electromagnetic waves and surface acoustic waves. The specific form of V_e^+ and V_a^+ depends on the device geometry and dielectric environment. In addition, V_a^+ depends on the piezoelectric coupling strength. In our design, V_a^+ is only significant at the electromechanical resonant frequency, while V_e^+ is not sensitive to frequency near the electromechanical resonance.

Because v_e and v_a are different, the two terms in Eq. S2 interfere constructively or destructively depending on ω

$$\begin{aligned}|V_2^-|^2 &= \left(V_e^+(\omega, V_1)e^{i\omega d/v_e} + V_a^+(\omega, V_1)e^{i\omega d/v_a}\right)\left(V_e^+(\omega, V_1)e^{-i\omega d/v_e} + V_a^+(\omega, V_1)e^{-i\omega d/v_a}\right)\\ &= (V_e^+)^2 + (V_a^+)^2 + 2V_e^+V_a^+\cos\left(\omega(d/v_e - d/v_a)\right),\end{aligned} \tag{S3}$$

which generates the oscillating features shown in Fig. 1e. Eq. S3 also shows that the oscillating frequency is proportional to d, consistent with the experimental results.

Extracting K^2 from microwave transmission coefficient

In this section, we discuss the circuit model of IDTs and the method used to extract the effective coupling coefficient K^2 from the microwave transmission coefficient S_{21}. The circuit model used is based on the classical analysis of IDT [1, 2]. Here, we give a brief introduction to it.

Fig. S6a and b show the circuit model of the loaded transmitter and receiver IDTs, respectively. The model of a single transducer includes the geometric capacitance C_g and a complex admittance $Y_a^{\mathrm{IDT}}(\omega) = G_a^{\mathrm{IDT}}(\omega) + iB_a^{\mathrm{IDT}}(\omega)$ that characterizes the electromechanical transduction. G_a^{IDT} and B_a^{IDT} are the real and imaginary parts of the effective admittance, respectively. At the electromechanical resonant frequency $f = f_0 = \omega_0/2\pi$, $B_a = 0$, and [3]

$$G_a^{\mathrm{IDT}}(\omega_0) = 8K^2\gamma C_g^{\mathrm{IDT}} f_0 N/\zeta. \tag{S4}$$

Here, N is the number of the IDT periodic units. γ and ζ are dimensionless parameters determined by the device geometry. For the split-finger transducers, $\gamma = 1.0836$ and $\zeta = 1.414$ [3].

During the measurement, the transmitter is connected to a voltage source with impedance $Z_0 = 50\ \Omega$. The energy consumed by G_a^{IDT} is converted to the mechanical energy of the surface acoustic waves. The receiver is driven by an effective voltage-controlled current source and is loaded by $50\ \Omega$. The energy consumed by the load represents the electrical energy converted from the mechanical power source. The insertion loss of the transmitter is

$$\mathrm{IL}_t = \frac{2G_a^{\mathrm{IDT}}Z_0}{(1+G_a^{\mathrm{IDT}}Z_0)^2 + [Z_0(\omega_0 C_g^{\mathrm{IDT}} + B_a^{\mathrm{IDT}})]^2}, \tag{S5}$$

which is defined as the ratio of the power converted into the power of the forward-propagating SAW to the maximal available power. The insertion loss of the receiver IL_r is the same as that of the transmitter by reciprocity. Therefore, the full microwave transmission coefficient is given by

$$|S_{21}(\omega_0)| = (\mathrm{IL}_t)^{1/2}\cdot L\cdot(\mathrm{IL}_r)^{1/2} = \frac{2G_a^{\mathrm{IDT}}Z_0L}{(1+G_a^{\mathrm{IDT}}Z_0)^2 + [Z_0(\omega_0 C_g^{\mathrm{IDT}} + B_a^{\mathrm{IDT}})]^2}, \tag{S6}$$

where L is the amplitude propagation loss during SAW propagation. Since $G_a^{\mathrm{IDT}} \ll 1/Z_0$, and $B_a^{\mathrm{IDT}} = 0$ at $f = f_0$,

$$|S_{21}(\omega_0)| \approx \frac{2G_a^{\mathrm{IDT}}(\omega_0)Z_0L}{1+(\omega_0 C_g Z_0)^2}, \tag{S7}$$

From Eq. S4 and S7, we obtain

$$K^2 = \frac{1+(\omega_0 C_g^{\rm IDT} Z_0)^2}{2Z_0}\frac{2\pi\zeta}{8\gamma C_g^{\rm IDT}\omega_0 NL}|S_{21}(\omega_0)|. \tag{S8}$$

Eq. S8 offers a way to calculate K^2 from the measured $S_{21}(\omega_0)$.

The above model ignores the capacitive crosstalk between the transmitter and receiver, which cannot be omitted when piezo-electricity is weak. Here, we employ the time-gating method to eliminate the contribution of capacitive crosstalk [4]. We begin by performing an inverse Fourier transform to convert the data into the time domain. Next, a bandpass filter is applied in the time domain to filter out the crosstalk contribution, which occurs much earlier than the SAW signal. Finally, we transform the filtered data back to the frequency domain. After removing the crosstalk contribution, the spectrum shows a clear maximum at the resonant frequency $f_0 = 4.583$ GHz. As shown in Fig. 2a, $|S_{21}(\omega_0)| = 1.1221\times10^{-5}$ or -99 dB.

From Fig. S7, the propagation loss at cryogenic temperature is negligibie. Therefore, $L = 1$. Substituting the calculated L into Eq. S8 with $N = 50$ and $C_g = 318$ fF, we obtained $K^2 \approx 3\times10^{-7}$.

Error analysis of $K^2_{\rm eff}$

The error in $K^2_{\rm eff}$ comes from both the noise of the detection circuits, and the uncertainties in frequency-dependent gain calibration of the microwave chain. The latter dominates the $K^2_{\rm eff}$ uncertainty.

The gain of the microwave chain was calibrated by measuring the averaged transmission coefficient S_{21} through a reference bypass coaxial cable between 4 GHz and 5 GHz. At 30mK, the voltage gain $G_{\rm mw} = 8.85\pm0.39$ where the variation is from dispersion caused by reflections. The error of $G_{\rm mw}$ can propagate to S_{21}, giving a relative uncertainty

$$\left(\frac{|\Delta S_{21}|}{|S_{21}|}\right)_g = \frac{\Delta G_{\rm mw}}{G_{\rm mw}} = 4.36\times10^{-2}. \tag{S9}$$

We use a Monte Carlo method to propagate the uncertainty to the time-gated S_{21} and therefore $K^2_{\rm eff}$. Suppose the expected value of measured $|S_{21}(f)|$ is $|S_{21,0}(f)|$. The relative error induced by gain calibration is $\sigma(f)$. The actually measured $S_{21}(f)$ is

$$|S_{21}(f)| = |S_{21,0}(f)|(1+\sigma(f)) \tag{S10}$$

The time-gated spectrum $S_{21,\rm TG}(f)$ can be calculated with error included. We represent σ with a random number obeying normal distribution with standard deviation of $\left(\frac{\Delta S_{21}}{S_{21}}\right)_n$, and repeat the above process for 5000 times to reach convergence. The uncertainty of $S_{21,\rm tg}(f)$ can be subsequently obtained by calculating the standard deviation of the results. At the resonant frequency, we obtain the relative uncertainty

$$\left(\frac{\Delta|S_{21,\rm TG}(f_0)|}{|S_{21,\rm TG}(f_0)|}\right) = 13.04\% \tag{S11}$$

Since $K^2_{\rm eff}$ is proportional to $|S_{21,\rm TG}(f_0)|$, the relative uncertainty of $k^2_{\rm eff}$ is

$$\left(\frac{\Delta K^2_{\rm eff}}{K^2_{\rm eff}}\right) = \left(\frac{\Delta|S_{21,\rm TG}(f_0)|}{|S_{21,\rm TG}(f_0)|}\right) = 13.04\% \tag{S12}$$

Qubit loss rate induced by the interface piezoelectricity

In this section, we derive the expression for the loss rate γ from the circuit model shown in Fig. 4a. It contains the circuit diagram of a transmon qubit with the interface-piezoelectricity-induced loss included. As discussed in the previous section, the piezoelectric effect can be represented by a parasitic radiation admittance $Y_a^q(\omega) = G_a^q(\omega) + iB_a^q(\omega)$, where G_a^q and B_a^q are the radiation conductance and susceptance, respectively. The total admittance between Node 1 and Node 2, shown in Fig. 4a, is

$$\begin{aligned} Y_{12}^q(\omega) &= \frac{1}{i\omega L_J} + i\omega C_g^q + G_a^q(\omega) + iB_a^q(\omega) \\ &= \frac{1-\omega^2 L_J[C_g^q + B_a^q(\omega)/\omega] + i\omega L_J G_a^q(\omega)}{i\omega L_J}. \end{aligned} \tag{S13}$$

Since the piezoelectricity is weak, the radiation susceptance B_a is negligible compared to ωC_g^q, i.e.,

$$Y_{12}^q(\omega) \approx \frac{1 - \omega^2 L_J C_g^q + i\omega L_J G_a^q(\omega)}{i\omega L_J}. \tag{S14}$$

Eq. S14 is the impedance of a parallel resonant circuit with resonant (plasmon) frequency

$$\frac{\omega}{2\pi} = \frac{1}{2\pi\sqrt{L_J C_g^q}} \tag{S15}$$

and quality factor

$$Q(\omega) = \frac{\omega C_g^q}{G_a^q(\omega)} = \frac{\omega}{\gamma(\omega)}, \tag{S16}$$

where $\gamma(\omega) = G_a^q(\omega)/C_g^q$ is the piezoelectric loss rate. The corresponding relaxation time of the resonator is given by

$$T_1(\omega_q) = \frac{1}{\gamma(\omega_q)} = \frac{C_g^q}{G_a^q(\omega_q)}, \tag{S17}$$

where $\omega_q = \omega_p - e^2/2C_g$ is transmon qubit frequency. Therefore, if $G_a^q(\omega_q)$ is known, $Q(\omega_q)$ or $T_1(\omega_q)$ can be obtained.

For the superconducting qubit with a shunt capacitor that has the same geometry as the IDTs studied in the experiment, G_a^q at the electromechanical resonance ($\omega = \omega_0$) can be obtained from S_{21} (Eq. S6). At other frequencies [2],

$$G_a^q(\omega) = G_a^q(\omega_0)\frac{[N\pi(\omega-\omega_0)/\omega_0]^2}{\sin^2[N\pi(\omega-\omega_0)/\omega_0]}. \tag{S18}$$

For the superconducting qubits with different (i.e., not IDT) geometries, $G_a^q(\omega)$ can be obtained numerically using the finite-element method. The first step is calculating $G_a^q(\omega)$ of an aluminum-on-silicon IDT with the same geometry used in the experiment. The piezoelectric coupling is simulated by assigning a finite e_{33} of a thin silicon layer with thickness h under the aluminum pattern that matches the measured $G_a^q(\omega_0)$. Different combinations of h and e_{33} can produce the same $G_a^q(\omega_0)$.Fig. S6d shows the four combinations we use for the qubit loss simulation. Then, we use finite-element multiphysics simulations to calculate the admittance of the desired qubit shunt capacitor with a piezoelectric silicon layer near the aluminum-silicon interface that has thickness h and piezoelectric coupling coefficient e_{33} using the values obtained. Fig. S10a and b show the geometry and dimensions of the capacitor models studied. The dimensions were chosen such that both geometries have a capacitance of 125 fF. These simulations give the $G_a^q(\omega)$ of the qubit shunt capacitor from which the qubit quality factor is calculated.

Supplementary table and figures

TABLE S1. Summary of the Samples Studied

Sample	Substrate	Fabrication Process	Measurement Environment	Figures
A	Silicon	Lift-off	Room temperature	1(e) - (g), 3(b) - (d), S2, S3(b), S4(a)
B	Silicon	Lift-off	Cryogenic	2(a) - (c), S3(c), S8(d) - (e), S8(g) - (l)
C	Silicon	Lift-off	Cryogenic	S7(a) - (d)
D	Aluminum Nitride on Silicon	Lift-off	Cryogenic	2(c) - (d), S8(d), S8(f) - (l)
E	Silicon	Lift-off, Oxide removed	Room temperature	3(b), 3(e) - (f), S4(b)
F	Silicon	Etch, Oxide removed	Room temperature	S4(c)
G	Silicon	Etch, Oxide removed	Cryogenic	S4(d)

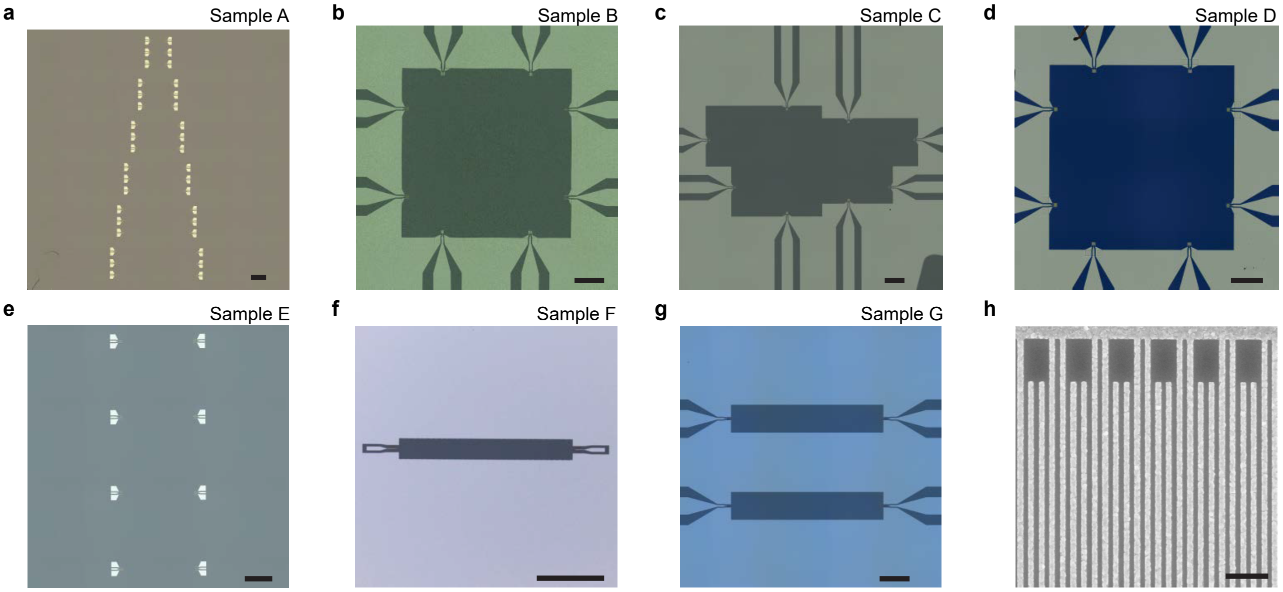

FIG. S1. **Optical and scanning electron micrographs of the studied samples. a** - **g**, Optical micrographs of Sample A, B, C, D, E, F, and G respectively. The scale bars represent 500 μm. **h**, Scanning electron micrograph of a split-finger transducer. The scale bar represents 1 μm.

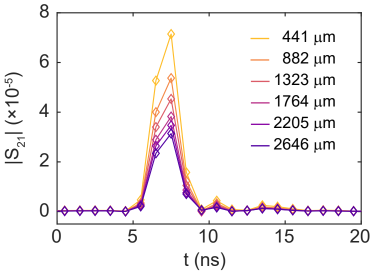

FIG. S2. **Distance-dependent crosstalk.** Zoom-in of Fig. 1e near the peak corresponding to the capacitive crosstalk. $|S_{21}|$ is plotted in linear scale for clarity. The peak transmission varies as a function of separation distance d, which explains the offset shown in Fig. 1e.

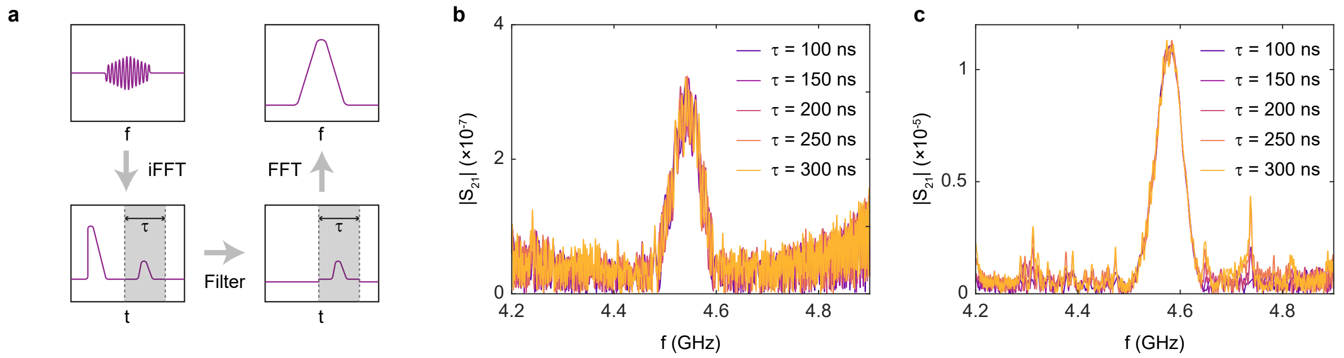

FIG. S3. **Time-gating procedure.** **a**, Schematic illustration of the time-gating process. The raw transmission coefficient S_{21} spectra were converted to the time-domain response by applying an inverse fast Fourier transform. The resulting trace typically contains two peaks: an early peak corresponding to capacitive crosstalk and a later one corresponding to surface acoustic wave–mediated transmission. A temporal bandpass filter with length τ was then applied to remove the early peak, followed by a forward fast Fourier transform to recover the time-gated S_{21} in the frequency domain. The amplitude of the data is shown here for clarity, though the actual processing was performed on the complex-valued S_{21}. **b**, Time-gated transmission coefficient measured at room temperature on Sample A using different time-window sizes τ. Linear scaling is used to show that the peak transmission amplitude is insensitive to the window size. **c**, Same as **b**, for Sample B measured at 30 mK. When a large time window is used, partial inclusion of the capacitive crosstalk peak produces sharp features away from the electromechanical resonance. Nevertheless, the peak transmission amplitude remains largely insensitive to the window size.

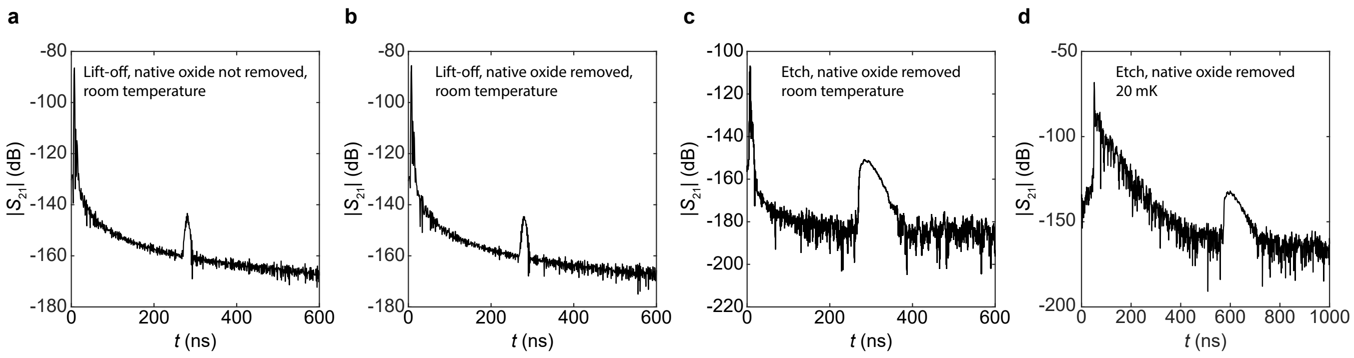

FIG. S4. **Interface piezoelectricity of samples fabricated with different processes.** **a**, Microwave transmission coefficient $|S_{21}|$ as a function of delay time t measured from Sample A (Fig. S1a) at room temperature. The sample was fabricated following an aluminum lift-off process. The native oxide was not removed before aluminum deposition. **b**, Microwave transmission coefficient $|S_{21}|$ as a function of delay time t measured from Sample E at room temperature. The sample was fabricated following an aluminum lift-off process. Native oxide was removed using buffered hydrofluoric acid before aluminum deposition. **c**, Microwave transmission coefficient $|S_{21}|$ as a function of delay time t measured from Sample F (Fig. S1f) at room temperature. The sample was fabricated by etching the pre-deposited aluminum film. Due to the limited pattern-transfer resolution, single-finger geometry was used for this sample. Such geometry does not suppress mass-loading-induced reflection of the surface acoustic waves as the transducers with split-finger geometry do; however, this does not affect the conclusion that interface piezoelectricity exists in etched samples. **d**, Microwave transmission coefficient $|S_{21}|$ as a function of delay time t measured from Sample G (Fig. S1g) at $T = 20$ mK. The sample was fabricated by etching the pre-deposited aluminum film.

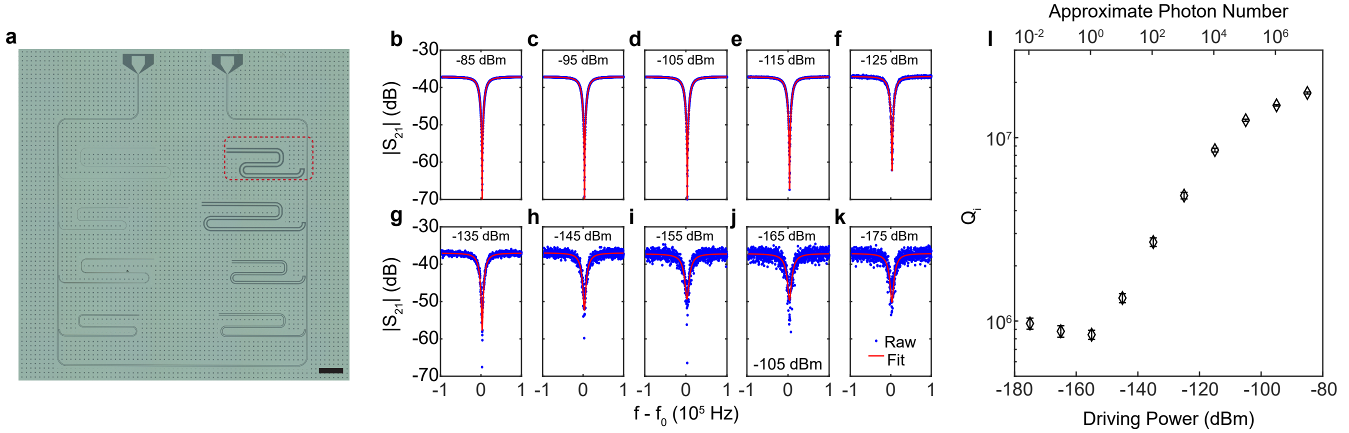

FIG. S5. **Characterizing the quality of aluminum-silicon interface using a coplanar waveguide resonator a**, Optical micrograph of the sample. The resonator being studied is marked with a red dashed rectangle and has a surface participation ratio of 4.5×10^{-4} [5]. The scale bar represents 500 μm. **b-k**, Microwave transmission coefficient of the device near the resonant frequency at different driving powers. Blue dots are the raw data. Red curves are fitting to a theoretical model. (See Ref. [5] for details) **l**, Internal quality factor Q_i as a function of driving power obtained by fitting the data in b, with fitting error indicated. The approximate photon numbers are labeled on the top axis.

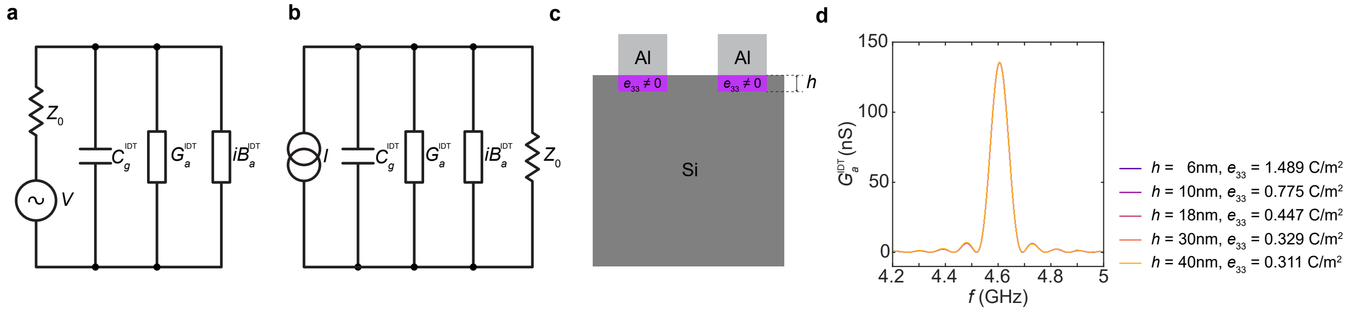

FIG. S6. **Circuit models of the IDTs**. **a**, Circuit model of the transmitter IDT. **b**, circuit model of the receiver IDT. **c**, Geometry for searching (h, e_{33}) combinations from experimentally determined K^2 using finite-element analysis. **d**, Simulated acoustic radiation admittance G_a^{IDT} of the IDT as a function of frequency. Four (h, e_{33}) combinations are found to produce nearly identical $G_a^{\mathrm{IDT}}(f)$, and therefore, nearly identical K^2.

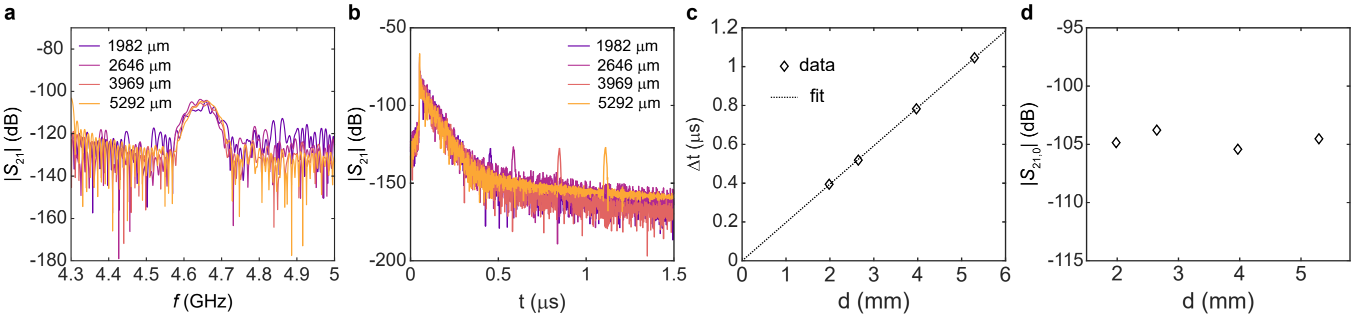

FIG. S7. **Measurement of propagation loss of surface acoustic waves at cryogenic temperature.** **a**, Time-gated microwave transmission coefficient near the electromechanical resonance measured from the four delay line IDTs with different separation distance d on Sample C (Fig. S1c) at $T = 20$ mK. No obvious attenuation is observed as d increases. **b**, Time-domain microwave transmission coefficient of the same devices in a. **c**, Diamonds: $\Delta t = t_s - t_c$ as a function of d. Here t_s (t_c) is the onset of transmission mediated by the surface acoustic waves (capacitive crosstalk). Dashed line: Linear fit $d = v \cdot \Delta t$ gives the surface acoustic wave velocity $v = 5065$ m/s. **d**, Microwave transmission coefficient at the electromechanical resonance as a function of d.

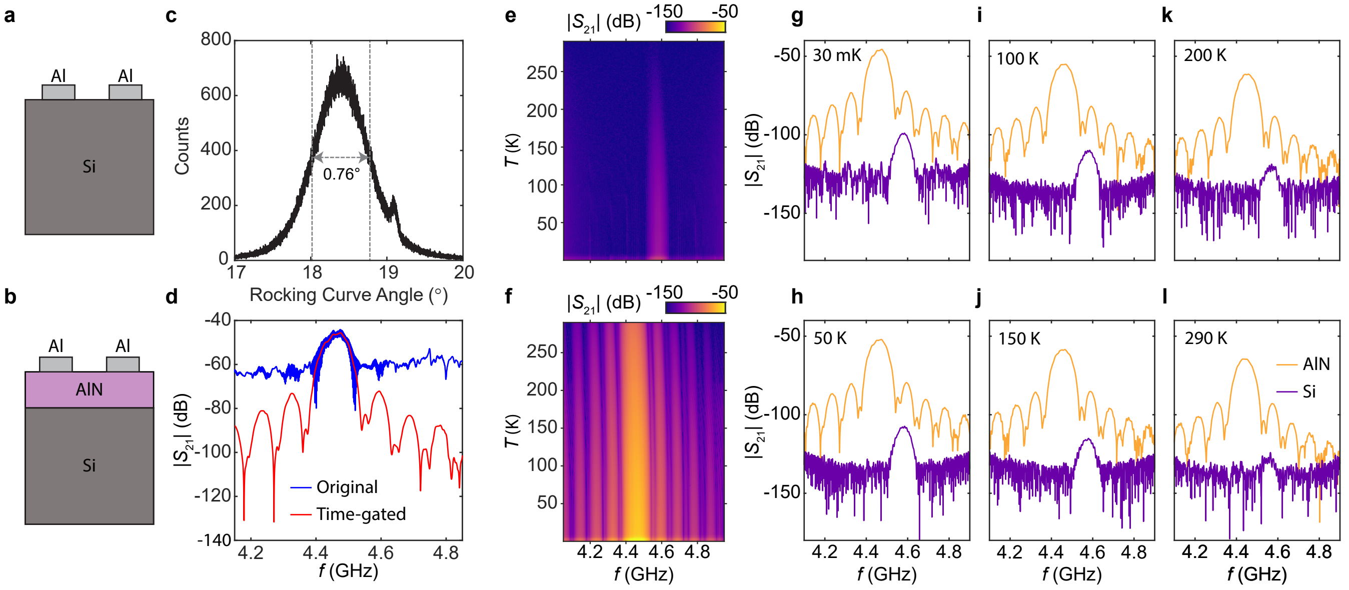

FIG. S8. **Comparison with IDTs fabricated on a piezoelectric aluminum nitride film.** **a**, Schematic cross-section of the aluminum-on-silicon surface acoustic wave transducers. **b**, Schematic cross-section of the aluminum-on-aluminum nitride surface acoustic wave transducer, used as a reference device (Sample D). **c**, X-ray diffraction rocking curve of the AlN film on silicon (111) substrate. The peak corresponds to the AlN (002) plane. The full width at the half maximum is measured to be 0.76°, smaller than 1°. The result indicates that the 33-component of the piezoelectric coefficient is close to that of single-crystal aluminum nitride [6–8]. **d**, Original and time-gated microwave transmission coefficient of Sample D (aluminum-on-aluminum nitride transducer) measured at $T = 30$ mK. **e**, Time-gated microwave transmission coefficient of Sample B directly read from the vector network analyzer. The gain of the microwave chain is not removed. **f**, Same as e, measured from Sample D. The same wiring was used to obtain results in panels e and f to ensure the gain of the microwave chain is the same. While dense temperature sampling was used, the temperature sampling points of panels e and f are not exactly the same. To compare the results and calculate the transmission coefficient difference shown in Fig. 2c, a linear interpolation of temperature was applied to the data from Sample D to match the temperature sampling point of measurement on Sample B. Then, the ratio of the microwave transmission coefficient of the two devices is calculated, shown in Fig. 2c. **g** - **l**, Comparison of time-gated microwave transmission coefficient between Sample B (aluminum-on-silicon transducer) and Sample D (aluminum-on-aluminum nitride transducer) at different temperatures indicated in the figure.

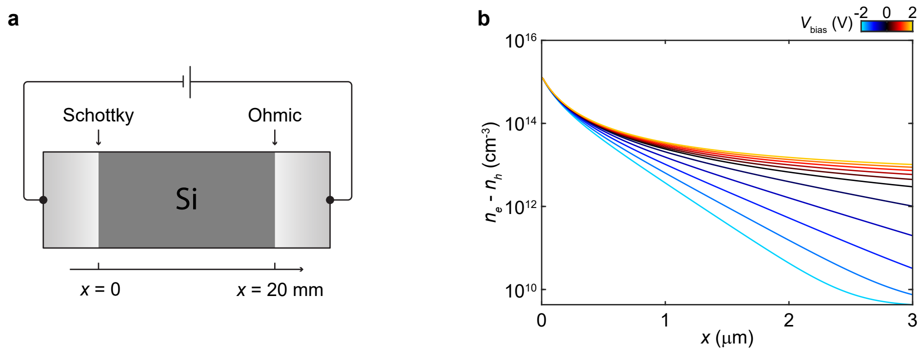

FIG. S9. **Simulated charge distribution in undoped silicon near the aluminum-silicon interface at room temperature using a finite element solver.** **a**, Schematic of the system studied. An ideal Ohmic contact is used to set the electrical potential on the other side of the silicon domain. **b**, Excess electron density $n_e - n_h$ as a function of x. Different curves correspond to different bias voltages applied across the structure. Here, n_e and n_h are the electron and hole densities, respectively. As expected, an accumulation layer is formed near the interface. The charge density near the interface increases when a positive voltage is applied on the back electrode and decreases when a negative voltage is applied.

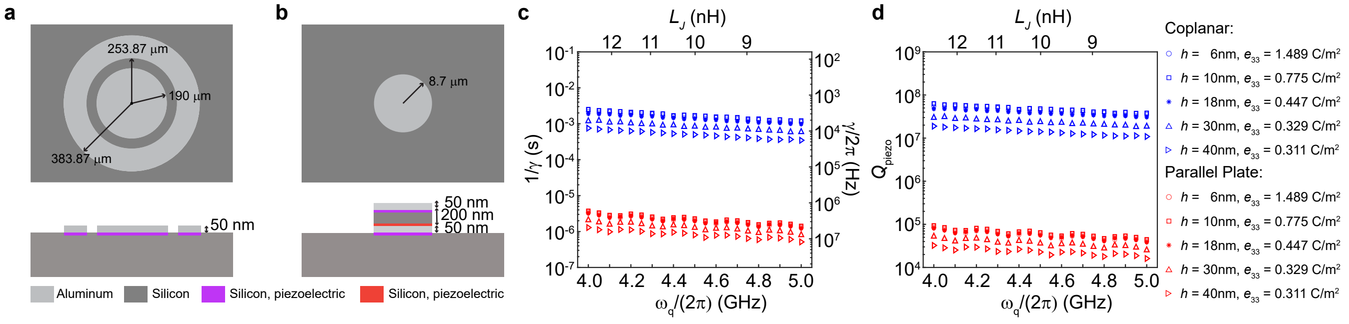

FIG. S10. **Details of interface piezoelectricity induced surface loss in superconducting qubits.** **a**, Top and cross-section view of the axial-symmetric coplanar capacitor. A piezoelectric region is added at the aluminum-silicon interface. The plots are not to scale. **b**, Same as panel a, for the axial-symmetric parallel-plate capacitor (PPC). The plots are not to scale. Since the direction of the electrical dipole moment near the interface between the upper aluminum plate and the silicon dielectric layer is opposite to that between the lower aluminum plate and the silicon dielectric layer, the signs of e_{33} for these two interfaces (rendered in purple and red, respectively) are opposite. **c**, Loss rate and interface-piezoelectric-loss-limited relaxation time of the transmon qubits as a function of the qubit frequency. Several different combinations of piezoelectric region thickness h and piezoelectric coupling coefficient e_{33} that produce the same K^2 are used for the calculation. **c**, Interface piezoelectric loss-limited quality factor Q_{piezo} as a function of the qubit frequency.

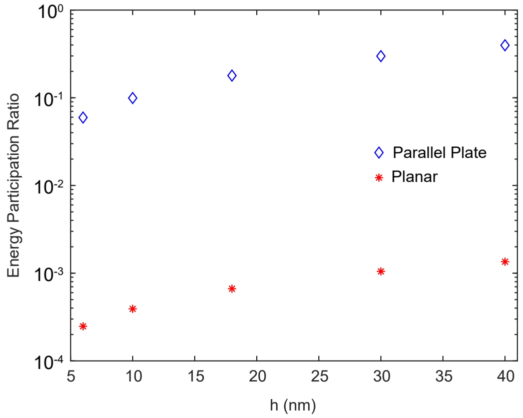

FIG. S11. **Energy participation analysis.** The ratio between the electrical energy stored within the effective piezoelectric layer near the aluminum-silicon interface and the total electric energy is calculated as a function of the effective piezoelectric layer thickness h.

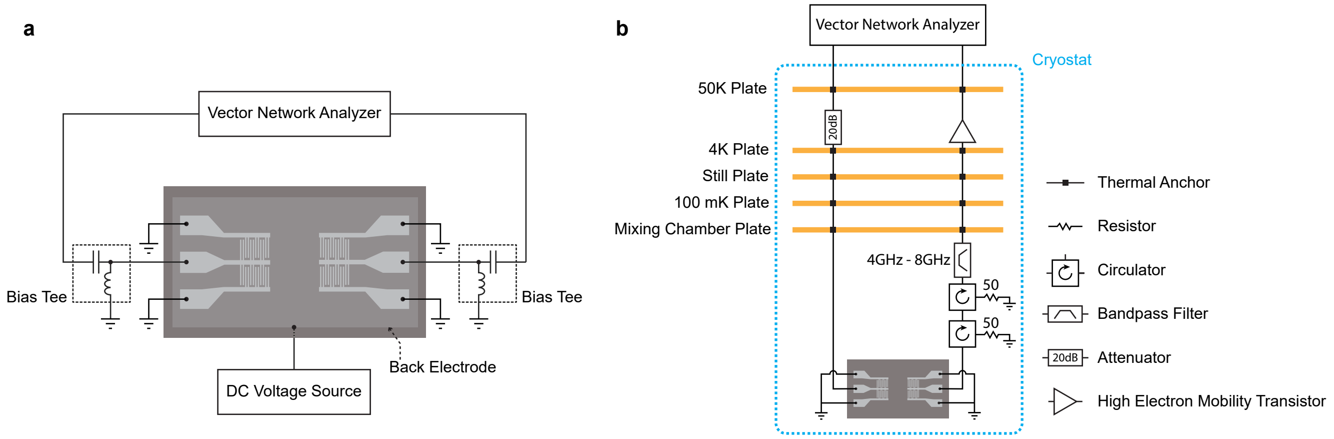

FIG. S12. **Wiring diagrams of the experiments.** **a**, Wiring diagram of the room temperature measurement. For samples without a back electrode, the bias tees are still included to ensure consistent background transmission, although the voltage is set to zero in this case. **b**, Wiring diagram of the cryogenic measurement.

* Present address: Department of Electrical Engineering and Computer Science, Massachusetts Institute of Technology, Cambridge, MA 02139

† Corresponding author: alp@berkeley.edu

[1] Smith, W., Gerard, H., Collins, J., Reeder, T. & Shaw, H. Analysis of interdigital surface wave transducers by use of an equivalent circuit model. *IEEE Trans. Microw. Theory Tech.* **17**, 856–864 (1969).

[2] Datta, S. *Surface Acoustic Wave Devices* (Prentice-Hall, 1986).

[3] Hines, J. H. & Malocha, D. C. A simple transducer equivalent circuit parameter extraction technique. In *1993 Proceedings IEEE Ultrasonics Symposium*, 173–177 vol.1 (1993).

[4] Yu, P. L. & Bhave, S. A. Acoustic delay lines to measure piezoelectricity in 4h silicon carbide. In *2017 Joint Conference of the European Frequency and Time Forum and IEEE International Frequency Control Symposium (EFTF/IFCS)*, 139–142 (2017).

[5] Zhang, Z.-H. *et al.* Acceptor-induced bulk dielectric loss in superconducting circuits on silicon. *arXiv e-prints* arXiv:2402.17155 (2024).

[6] Naik, R. S. *et al.* Measurements of the bulk, c-axis electromechanical coupling constant as a function of aln film quality. *IEEE Trans. Ultrason. Ferroelectr. Freq. Control* **47**, 292–296 (2000).

[7] Tonisch, K. *et al.* Piezoelectric properties of polycrystalline aln thin films for mems application. *Sens. Actuators A: Phys.* **132**, 658–663 (2006).

[8] Mishin, S. *et al.* Sputtered aln thin films on si and electrodes for mems resonators: relationship between surface quality microstructure and film properties. In *IEEE Symposium on Ultrasonics, 2003*, vol. 2, 2028–2032 Vol.2 (2003).
